# Responses of photosynthetic parameters to drought in subtropical forest ecosystem of China

**DOI:** 10.1038/srep18254

**Published:** 2015-12-15

**Authors:** Lei Zhou, Shaoqiang Wang, Yonggang Chi, Qingkang Li, Kun Huang, Quanzhou Yu

**Affiliations:** 1 Key Laboratory of Ecosystem Network Observation and Modelling, Institute of Geographic Sciences and Natural Resources Research, Chinese Academy of Sciences, Beijing 100101, China; 2 State Key Laboratory of Vegetation and Environmental Change, Institute of Botany, Chinese Academy of Sciences, Beijing 100093, China

## Abstract

The mechanism underlying the effect of drought on the photosynthetic traits of leaves in forest ecosystems in subtropical regions is unclear. In this study, three limiting processes (stomatal, mesophyll and biochemical limitations) that control the photosynthetic capacity and three resource use efficiencies (intrinsic water use efficiency (iWUE), nitrogen use efficiency (NUE) and light use efficiency (LUE)), which were characterized as the interactions between photosynthesis and environmental resources, were estimated in two species (*Schima superba* and *Pinus massoniana*) under drought conditions. A quantitative limitation analysis demonstrated that the drought-induced limitation of photosynthesis in *Schima superba* was primarily due to stomatal limitation, whereas for *Pinus massoniana,* both stomatal and non-stomatal limitations generally exhibited similar magnitudes. Although the mesophyll limitation represented only 1% of the total limitation in *Schima superba*, it accounted for 24% of the total limitations for *Pinus massoniana*. Furthermore, a positive relationship between the LUE and NUE and a marginally negative relationship or trade-off between the NUE and iWUE were observed in the control plots. However, drought disrupted the relationships between the resource use efficiencies. Our findings may have important implications for reducing the uncertainties in model simulations and advancing the understanding of the interactions between ecosystem functions and climate change.

Water deficit is the primary factor that limits ecosystem productivity in most terrestrial biomes^1^. The physiological responses of trees to drought (i.e., carbon uptake) are directly related to vegetation growth^2^, ecosystem productivity^3^,^4^, frequency of fires^5^,^6^ and tree mortality^7^,^8^. The subtropical region experiences frequent seasonal droughts^9^ that result in declines in terrestrial carbon sequestration^10^. However, the mechanism underlying the effects of drought on the carbon uptake of subtropical ecosystems at the leaf level remains unclear^11^.

The carbon uptake of forest ecosystems is driven by leaf photosynthesis, the responses of which to drought are mediated by three physiological processes. First, stomatal closure is recognized as the main driver of the photosynthetic response to water stress by limiting CO_2_ diffusion from the atmosphere to the substomatal cavities to slow photosynthesis^12^,^13^. Second, the mesophyll conductance (*g*_m_) may rapidly decrease, thereby limiting CO_2_ diffusion from the substomatal cavities to the chloroplast stroma during water stress^14^,^15^. Finally, photosynthesis may be limited by biochemical processes in long-lasting, severe droughts, resulting in decreased photosynthetic enzyme activity (i.e., the maximum rate of Rubisco carboxylation, *V*_cmax_), ribulose-1,5-bisphophate (RuBP) regeneration capacity (i.e., the maximum rate of photosynthetic electron transport, *J*_max_) and triose-phosphate utilization (TPU)^16^,^17^,^18^. As a result, drought stress directly influences CO_2_ diffusion and/or the biochemical process of photosynthesis, which in turn limits the net CO_2_ assimilation rate (*A*_n_). For example, Maseda and Fernandez (2006) found that the rapid closure of stomata during water stress resulted in a decline in transpiration and the *A*_n_^19^. Increasing evidence has shown that mesophyll conductance is finite^20^ and plays an important role in limiting the photosynthetic capacity^12^. Additionally, drought-stressed plants exhibit significant reductions in *V*_cmax_, *J*_max_ and *TPU* relative to plants with sufficient water^21^, indicating that biochemical processes dramatically inhibit photosynthesis during long-term severe droughts. These apparent discrepancies may arise from the fact that photosynthesis induced by drought stress is not limited by a single process. Instead, the combined effect of the stomatal, mesophyll, and biochemical limitations simultaneously regulates the decrease in photosynthesis in response to water stress^21^,^22^,^23^. However, a quantitative limitation analysis is needed to separate the three physiological processes in subtropical climatic zones^12^,^22^,^24^.

Drought not only decreases the leaf photosynthetic rate but also regulates the interaction of plant carbon uptake and environmental resources, which is termed the resource use efficiency. Three resource use efficiencies (water use efficiency (WUE), carbon gain at the expense of water loss; nitrogen use efficiency (NUE), carbon gain per nitrogen content; and light use efficiency (LUE), carbon gain per available light quantum flux density) are important functional parameters that intimately couple the uptake of carbon with the major growth limiting factors (water, nitrogen and light). In general, the leaf-level WUE has been reported to increase during soil water stress^25^,^26^, which suggests that stomata closure in response to H_2_O flux is more sensitive than the response to carbon flux^27^. Apart from increasing the WUE, stomatal closure during drought stress has an effect on photosynthesis but no effect on leaf nitrogen, leading to a decline in the NUE^26^,^27^. During drought stress periods, the LUE generally decreases with increasing drought intensity; indeed, no change was observed in the electron transport rate under mild and moderate water stress^28^, or the electron transport rate declined to a lesser extent than the net CO_2_ assimilation rate^29^. The changes in single resource use efficiency induced by drought have been well documented^25^,^26^,^28^, but the trade-off among the multiple resource use efficiencies of plants requires investigation.

Based on a recent integration of eddy covariance observations, subtropical forests in the East Asian region exhibit a high carbon dioxide uptake rate (362 g C m^−2^ year^−1^) compared with Asian tropical and temperate forests^30^. A model simulation indicated that drought caused the net exchange of carbon in the subtropical forests in Southern China to decrease by 63% and 47% in 2003 and 2004, respectively^31^. Despite the ecological importance of this region, the carbon uptake response of subtropical forests to drought is poorly constrained. To address this knowledge gap, a rainfall exclusion experiment was established in 2010 using two gradients of soil moisture content and three replicates. The three limiting processes (i.e., stomatal limitation, mesophyll limitation and biochemical limitation) that control photosynthesis and the three resource use efficiencies (iWUE, NUE and LUE) that represent the interaction of photosynthesis and environmental resources were estimated after a 3-year drought. We focused on (1) testing the sensitivity of the photosynthetic characteristics of *Schima superba* and *Pinus massoniana* in subtropical regions of China during an experimental drought and (2) determining the changes in the iWUE, NUE and LUE in response to water stress and identifying whether a trade-off existed among resource use efficiencies.

## Results

### The response of soil water content and leaf chemical characteristics to drought

The experimental drought significantly reduced the soil water content by 38% (*t* = 9.840; *P* < 0.0001) (Table 1). In general, the leaf traits (i.e., SLA, C concentration, N concentration and C/N ratio) for each species were not affected by the drought based on an independent sample T-test, except that a significant difference in the C/N ratio for *Schima superba* (*P* = 0.044) occurred between the control and drought plots (Table 1).

### The response of the carbon assimilation process to drought

**Figure 1:**
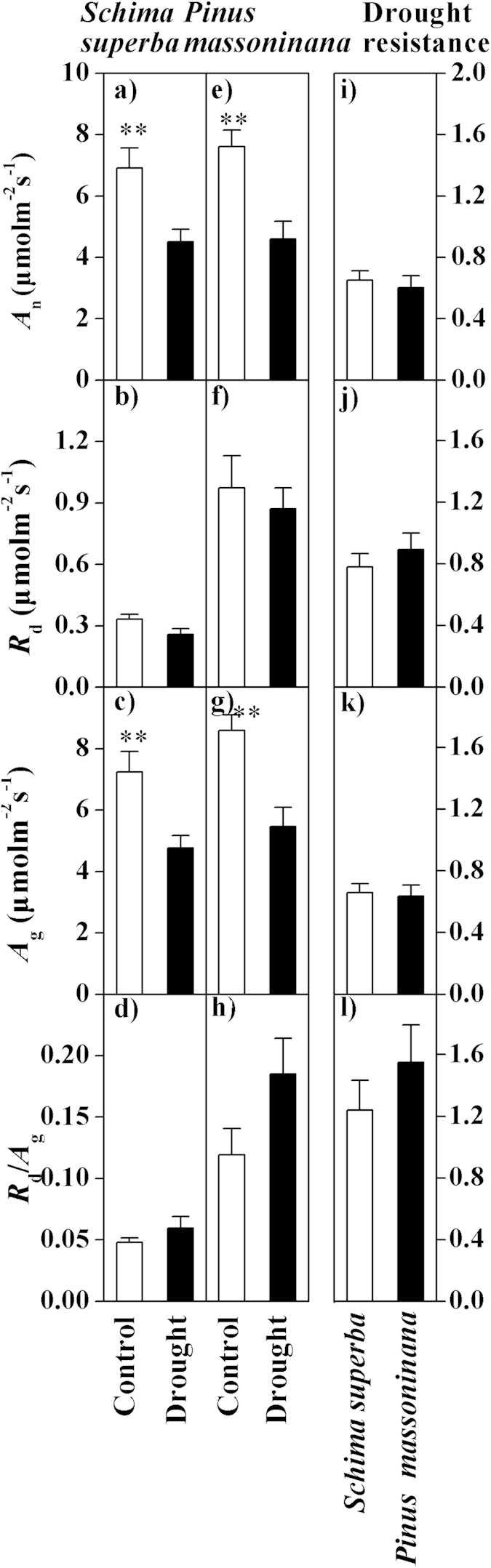
The effect of drought on the carbon assimilation process for the two species. (**a**,**e**) The *A*_n_ (net CO_2_ assimilation rate, μmol CO_2_ m^−2^ s^−1^), (**b**,**f**) *R*_d_ (day respiration, μmol CO_2_ m^−2^ s^−1^), (**c**,**g**) *A*_g_ (gross CO_2_ assimilation, μmol CO_2_ m^−2^ s^−1^) and (**d**,**h**) ratio of *R*_d_ and *A*_g_ in the control and drought plots for *Schima superba* (**a–d**) and *Pinus massoniana* (**e**–**h**) are shown. The drought resistance of (**i**) *A*_n_, (**j**) *R*_d_, (**k**) *A*_g_ and (**l**) *R*_d_/*A*_g_ in *Schima superba* and *Pinus massoniana* is indicated. ANOVA: *P < 0.05; **P < 0.01; and ***P < 0.001.

Based on an independent sample T-test, significant effects of drought on the *A*_n_ of *Schima superba* (*t* = 3.080, *P* = 0.005) and *Pinus massoniana* (*t* = 3.769, *P* = 0.001) were observed, which showed significant reductions during drought treatment (Fig. 1a,e). However, no significant effects of soil moisture on the *R*_d_ were observed (both *P* > 0.05) for either species (Fig. 1b,f). For *Schima superba* and *Pinus massoniana*, the responses of the *A*_g_ to the soil water treatments were significant (*t* = 3.134, *P* = 0.005 and *t* = 3.867, *P* = 0.001) and resulted in significant decreases in the drought plots compared with the control plots (Fig. 1c,g). Although there were no significant differences in *R*_d_/*A*_g_ between drought treatments for each species, a general increasing trend from the control to the drought plots was indicated (Fig. 1d,h). No significant differences in drought resistance for *A*_n_, *R*_d_, *A*_g_, and *R*_d_/*A*_g_ were observed between *Schima superba* and *Pinus massoniana* (Fig. 1i–l).

### The response of the CO_2_ diffusion process to drought

**Figure 2:**
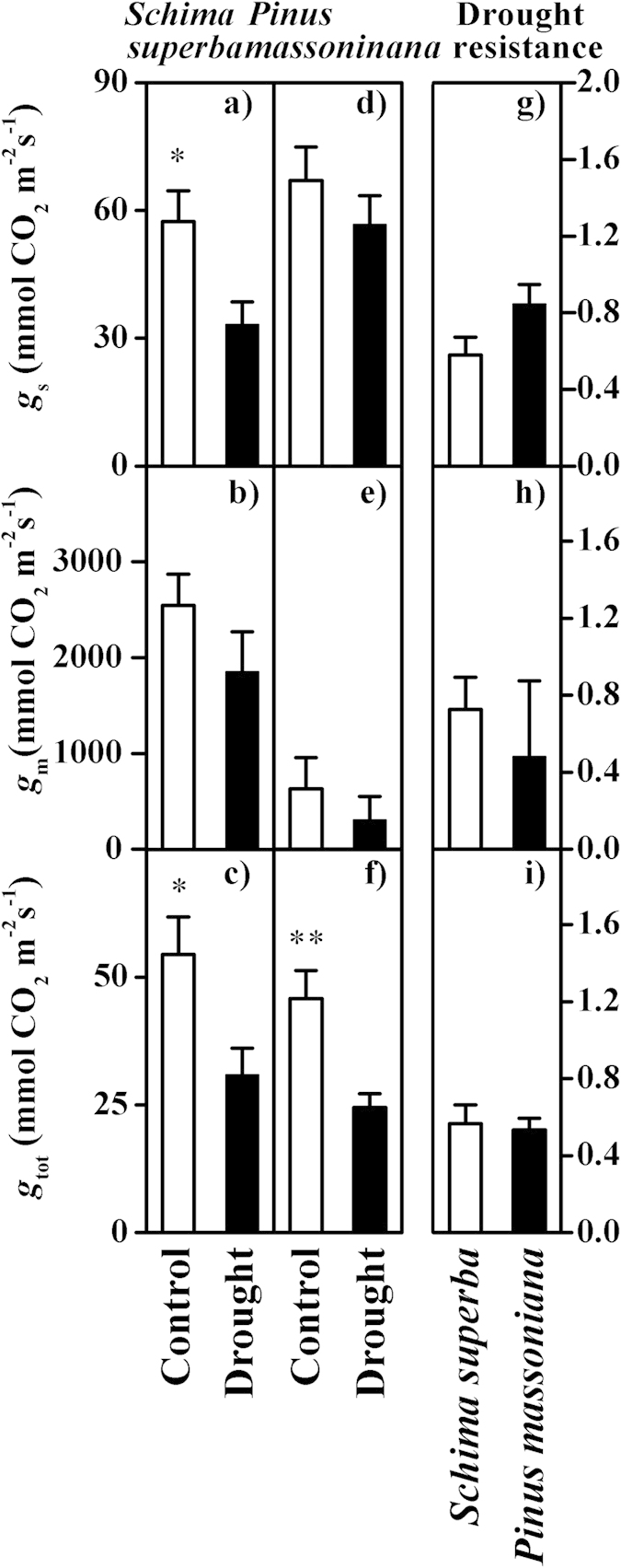
The effect of drought on the CO_2_ diffusion process for the two species. (**a**,**d**) The *g*_s_ (stomatal conductance, mmol CO_2_ m^−2^ s^−1^), (**b**,**e**) *g*_m_ (mesophyll conductance, mmol CO_2_ m^−2^ s^−1^) and (**c**,**f**) *g*_tot_ (the total conductance, mmol CO_2_ m^−2^ s^−1^) in the control and drought plots of *Schima superba* (**a**–**c**) and *Pinus massoniana* (**d**–**f**) are shown. The drought resistance of (**g**) *g*_s_, (**h**) *g*_m_ and (**i**) *g*_tot_ in *Schima superba* and *Pinus massoniana* is indicated. ANOVA: *P < 0.05; **P < 0.01; and ***P < 0.001.

Drought produced a 42% decrease in the *g*_s_ (*t* = 2.709, *P* = 0.013) for *Schima superba* (Fig. 2a), whereas the effect of drought on the *g*_s_ for *Pinus massoniana* was not significantly different between the control and drought plots (*P* > 0.05) (Fig. 2d). The responses of the *g*_m_ to drought were not significantly different for either species, based on an independent sample T-test (both *P* > 0.05) (Fig. 2b,e). However, significant decreases in the *g*_tot_ were observed (*Schima superba*: *t* = 2.618, *P* = 0.016; *Pinus massoniana*: *t* = 3.583, *P* = 0.002) in the drought plots relative to the control plots (Fig. 2c,f). The drought resistance of *Pinus massoniana* with regard to the *g*_s_ appeared to be considerably higher than that of *Schima superba* (*P* = 0.065), whereas no significant differences in drought resistance related to the *g*_m_ and *g*_tot_ were found between the species (Fig. 2g–i).

### The response of biochemical processes to drought

The effects of drought on *V*_cmax_ and *J*_max_ were not significantly different between the control and drought plots for *Schima superba* and *Pinus massoniana* (all *P* > 0.05, Figure S1a-b, e-f). For *Schima superba*, a significant increasing trend in *J*_max_/*V*_cmax_ (*t* = −2.229, *P* = 0.036) was observed from the control to the drought plots, whereas no significant effect of drought on *J*_max_/*V*_cmax_ was observed for *Pinus massoniana* (*P* > 0.05). No significant differences in drought resistance were observed for *V*_cmax_, *J*_max_, TPU and *J*_max_/*V*_cmax_ between *Schima superba* and *Pinus massoniana* (Figure S1i-l).

### Quantitative limitation analysis

**Figure 3:**
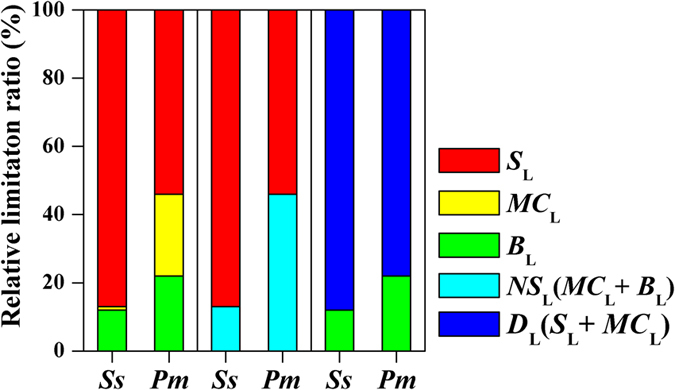
The quantitative limitation of *A*_n_ (net CO_2_ assimilation rate, μmol CO_2_ m^−2^ s^−1^) for *Schima superba* (*Ss*) and *Pinus massoniana* (*Pm*) during drought stress. The stomatal limitation (*S*_L_, red area), mesophyll conductance limitation (*MC*_L_, yellow area) and biochemical limitation (*B*_L_, green area) are shown. The total diffusional limitation (*D*_L_ = *S*_L_ + *MC*_L_, blue area) and the non-stomatal limitation (*NS*_L_ = *MC*_L_ + *B*_L_, sky blue area) are also indicated.

For *Schima superba,* the values of *S*_L_, *MC*_L_ and *B*_L_ accounted for 87%, 1% and 12% of the limitations, respectively (Fig. 3). The contributions of the stomatal (*S*_L_) and non-stomatal limitations (*NS*_L_ = *MC*_L_ + *B*_L_) represented approximately seven-eighths and one-eighth of the total limitation, respectively. The role of the total diffusional limitation (*D*_L_ = *S*_L_ + *MC*_L_) was more important than that of the biochemical (*B*_L_) limitation.

For *Pinus massoniana*, the *S*_L_, *MC*_L_ and *B*_L_ values were equal to 54%, 24%, and 22% of the limitations, respectively. The stomatal (*S*_L_) and non-stomatal limitations (*NS*_L_) generally showed a similar magnitude. The contributions of the total diffusional limitation (*D*_L_) and biochemical limitation (*B*_L_) represented approximately two-thirds and one-third of the total limitation, respectively.

### Resource use efficiency

**Figure 4:**
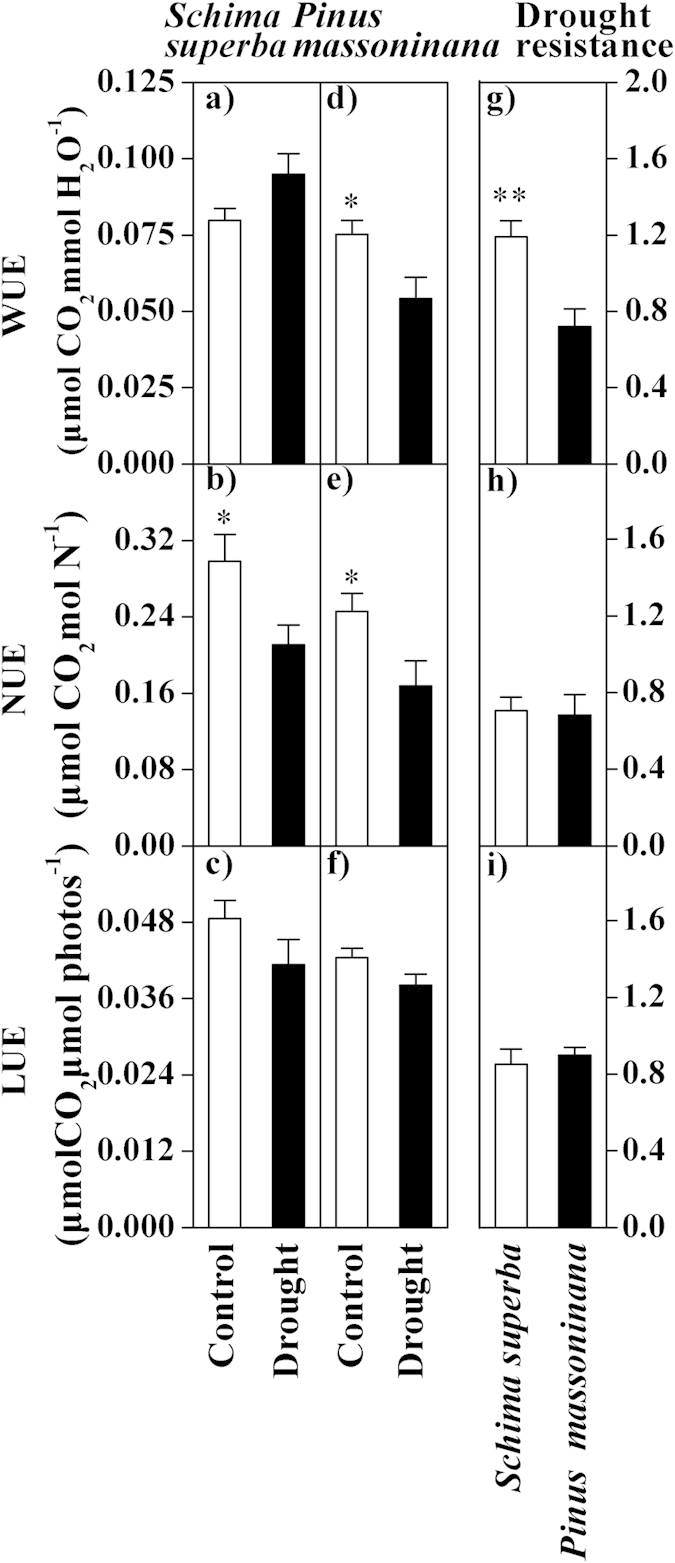
The effect of drought on the resource use efficiency for the two species. (**a**,**d**) The iWUE (the water use efficiency, μmol CO_2_ mmol H_2_O^−1^), (**b**,**e**) NUE (the nitrogen use efficiency, μmol CO_2_ mol N^−1^) and (**c**,**f**) LUE (the light use efficiency, μmol CO_2_ μmol Photons^−1^) in the control and drought plots of *Schima superba* (**a**–**c**) and *Pinus massoniana* (**d**–**f**) are shown. The drought resistance of the (**g**) iWUE, (**h**) NUE, and (**i**) LUE in *Schima superba* and *Pinus massoniana* is indicated. ANOVA: *P < 0.05; **P < 0.01; and ***P < 0.001.

A general increasing trend the in iWUE from the control to the drought plots was observed for *Schima superba* (*P* = 0.056), whereas significant declines in the iWUE due to the drought treatments (*P* = 0.021) were observed for *Pinus massoniana* (Fig. 4a,d). The effects of drought on the NUE of *Schima superba* and *Pinus massoniana* exhibited significant reductions from the control to drought plots (Fig. 4b,e). No significant differences were observed in the LUE for *Schima superba* and *Pinus massoniana* during drought treatments (all *P* > 0.05, Fig. 4c,f). The drought resistance of the iWUE exhibited significant differences between species, whereas no significant differences in drought resistance related to the LUE and NUE were observed between species (Fig. 4g-i).

**Figure 5:**
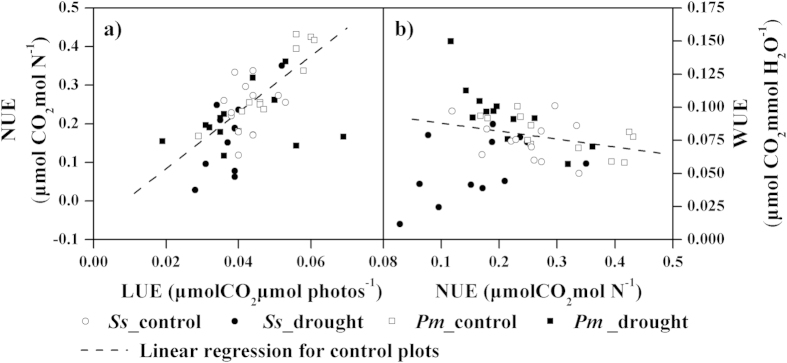
The relationships between the LUE (light use efficiency, μmol CO_2_ μmol Photons^−1^) and NUE (nitrogen use efficiency, μmol CO_2_ mol N^−1^) (a) or the NUE and iWUE (water use efficiency, μmol CO_2_ mmol H_2_O^−1^) (b) in the control and drought plots. *Ss* and *Pm* represent *Schima superba* and *Pinus massoniana*, respectively. The dotted line indicates the linear regression for the control plots.

To determine the trade-off in resource use efficiency, simple linear regressions of LUE vs. NUE and NUE vs. iWUE were performed (Fig. 5). A significant positive correlation was found between the LUE and NUE in the control plots (*y* = 7.29x−0.06, *R*^2^ = 0.57, *P* < 0.0001). However, poor correlations between the LUE and NUE were found for all species in the drought plots (*R*^2^ = 0.06, *P* = 0.237). As a result, the regression slopes of the LUE and NUE were different between the control and drought treatments (*P* = 0.034). A marginally negative relationship between the NUE and iWUE was observed for all species in the control plots (*y* = −0.06x + 0.09, *R*^2^ = 0.12, *P* = 0.092), whereas the correlations between the NUE and iWUE were weak (*R*^2^ = 0.02, *P* = 0.481) in the drought plots. However, no significant difference was observed in the regression slopes of the NUE and iWUE for the control and drought treatments (*P* = 0.179).

## Discussion

### Quantitative limitation analysis of photosynthesis in response to drought

As expected, drought stress significantly decreased the leaf photosynthesis of the dominate species (*Schima superba* and *Pinus massoniana*) in subtropical forests. Our study reported that an approximately 37% decrease in the *A*_n_ in the drought plots was related to a decrease of approximately 38% in the SWC. The pattern of decreasing *A*_n_ with drought was similar to the pattern observed in forests under field conditions^24^,^32^,^33^. However, the degree to which drought affected the *A*_n_ did not significantly vary between *Schima superba* and *Pinus massoniana* (Fig. 1i). Two independent experiments on mesic and xeric species from diverse hydroclimates in Australia and Europe indicated interspecific differences in the drought response^23^. The velocity of the photosynthetic changes in response to water stress imposition were faster in evergreen forests than in semi-deciduous forests, although the declines in photosynthetic rate were similar in magnitude^34^. Although water stress is known to reduce the photosynthetic rate, the processes responsible for the key limitations are still a matter of debate^35^. Previous studies have demonstrated that the photosynthetic reaction to water stress is dominated by only one of the three physiological processes (stomatal conductance, mesophyll conductance and biochemical processes)^36^,^37^,^38^,^39^. Increasing evidence had shown that the combined effect of the stomatal, mesophyll, and biochemical limitations simultaneously regulates the decline in photosynthesis in response to drought. Thus, a quantitative limitation analysis of the changes in the photosynthetic rate in response to water stress was necessary^12^,^40^,^41^. Our quantitative limitation analysis demonstrated that the drought-induced limitation of photosynthesis in *Schima superba* was mainly due to the stomatal limitation (87%), whereas the stomatal (*S*_L_, 54%) and non-stomatal limitations (*NS*_L_, 46%) for *Pinus massoniana* generally showed similar magnitudes. A study of temperate deciduous forests reported a maximum value of 50% for the *S*_L_ during drought stress^39^. A study of tropical evergreen forests (*Campsiandra laurifolia*, *Symmeria paniculata*, *Acosmium nitens* and *Eschweilera tenuifolia*) indicated that the *S*_L_ accounted for 30–39% of the limitations in the dry season (March 2004)^42^. Therefore, the magnitude of the photosynthetic reaction to water stress for the three types of limitations varied between species.

Mesophyll conductance is typically absent in gas exchange measurements, which are assumed to be infinite^43^. However, studies have demonstrated that changes in mesophyll conductance in response to stress and limit photosynthesis are an important physiological process^12^,^44^. For a variety of climate zones and species, the *MC*_L_ is responsible for approximately 14-30% of the limitations^12^,^22^,^23^,^24^. Similarly, Grassi and Magnani (2005) found a maximum value of 14% for the *MC*_L_ for an ash forest under seasonal drought^12^. Another rainfall exclusion experiment in a *Quercus ilex* forest showed a maximum value of 20% for the *MC*_L_^22^. Our quantitative limitation analysis showed that the *MC*_L_ was responsible for only 1% of the total limitation of *Schima superba*, although this limitation accounted for 24% of the total limitation for *Pinus massoniana*. Therefore, it is important to include mesophyll conductance into any detailed study of the gas exchange response to drought and the processed model.

An explanation for the discrepancies in the *MC*_L_ between both species in the subtropical region may be related to the different phylogenetic clades (gymnosperms vs. angiosperms)^45^. The SLA was reported to strongly separate gymnosperms from angiosperms based on 305 North American woody species that spanned boreal to subtropical climates^46^. The *g*_m_ can be influenced by changes in leaf anatomical characteristics, such as the thickness of leaf/mesophyll cell walls/chloroplasts and the stomata density^47^,^48^,^49^. Gymnosperms have a lower SLA value^50^, lower mesophyll porosity, thicker mesophyll cell wall^51^, and lower *g*_m_^51^,^52^ than angiosperms. In our study, the gymnosperm species (*Pinus massoniana*) had lower SLA and *g*_m_ values than angiosperms, which might contribute to the high *MC*_L_ in evergreen conifers.

### The balance between J_max_ and V_cmax_

The *J*_max_ and *V*_cmax_ relationship represents the resource allocation between the two photosynthetic cycles: electron transport and the Calvin cycle^53^. In the biochemically-based photosynthesis model, *V*_cmax_ was scaled to *J*_max_ based on the hypothesis that the average *J*_max_/*V*_cmax_ ratio was 2.1^54^. However, some studies have demonstrated that the *J*_max_ to *V*_cmax_ ratio is not constant but varies with temperature^55^, leaf nitrogen^56^, and species^57^. The underlying processes responsible for the changes in the *J*_max_/*V*_cmax_ ratio due to water stress are still a matter of debate. We found that the *J*_max_/*V*_cmax_ ratio varied considerably among drought treatments in *Schima superba* (Fig. S1d), which was consistent with previous studies^24^,^58^. The hypothesis that droughts modify the balance between RuBP carboxylation and regeneration was supported by our study (i.e., the resource allocation between the two photosynthetic cycles (the Calvin cycle and electron transport) was changed). Current ecosystem models are less capable of accounting for climate extremes (warming, nitrogen deposition or drought), which attributes to model calibration using data collected under standard climate conditions^59^. The findings from manipulation experiments could reduce the uncertainties of model parameter estimates and the predictions made by the models.

### The trade-off between resource use efficiencies (iWUE, NUE and LUE)

In the present study, we found a significant positive correlation between the LUE and NUE for all species in the control plots (Fig. 5a). Generally, the plants tended to obtain the maximum NUE with an increase in the LUE^60^. However, a poor correlation was observed between the LUE and NUE in the drought plots, indicating that drought stress might have weakened the relationship between the LUE and NUE in these plots (Fig. 5a). The slopes of the regression lines (LUE vs. NUE) from the control and drought plots were significantly different (*P* = 0.034). Furthermore, the NUE but not the LUE was significantly decreased in the drought treatments for each species (Fig. 4b,c,e,f). These results suggested that the responses of the LUE and NUE to drought were uncoupled. The use of resources (light and nitrogen) for plants may also be influenced by other factors, such as water stress.

Additionally, a marginally negative correlation was observed between the NUE and iWUE in the control plots for the two species studied here, which provided evidence for the existence of a trade-off between the uses of resources. An increase in the NUE together with a decrease in the iWUE indicated that compromises existed between the iWUE and NUE. This finding was consistent with previous studies of a large number of plants species^61^,^62^,^63^. An increase in the efficiency of the use of one resource can lead to a reduction in the efficiency of use of another resource^60^, suggesting that maximization of resource use efficiency depends on the most limited resources^33^. This trade-off may help enforce the species distribution across moisture and nutrient gradients^62^.

## Methods

### Experimental site

The experimental site was located in the Qianyanzhou (QYZ) Forest Experimental Station (26°44′N, 115°03′E) in Jiangxi Province of South China and belongs to Chinaflux. The mean annual air temperature is approximately 17.9 °C, and the highest and lowest daily temperatures recorded are 39.5 and −5.8 °C, respectively^64^. The annual average precipitation is 1,489 mm and mainly occurs from March to June (52% of total). The annual air temperature and annual precipitation in 2012 were 18.5 °C and 1741 mm, respectively. These characteristics are typical of the prevailing subtropical monsoon climate. The soil mainly consists of the red soil type formed principally from red sandstone, sand gravel or mudstone and river alluvial deposits. The main forest types include Masson’s pine (*Pinus massoniana*), slash pine (*Pinus elliottii*), Chinese fir (*Cunninghamia lanceolata*), and Schima (*Schima superba*). *Pinus massoniana* and *Schima superba* are the pioneer species and the dominant species in this region, respectively. Both species were mixed as conifer-broadleaf forests, with a tree density of stems of 700 ha^−1^.

### Rainfall exclusion experiment

**Figure 6:**
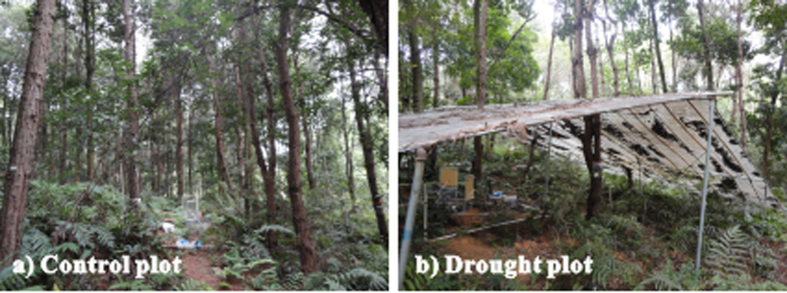
Rainfall exclusion experiments at the QYZ station: (a) Control plot and (b) Drought plot. Photo credit: Lei Zhou.

The rainfall exclusion experiment was initiated in January 2010. Rainfall was withheld for the entire year with no changes in other meteorological variables (Fig. 6). We used transparent polyester film placed at a height of 2 m on the trees to partially exclude throughfall drops onto the floor and allow light penetration. Trenches were dug around the perimeters of the plots to reduce the lateral inflow of water from the surrounding forest into the plots. Three rainfall exclusion plots (12 m × 12 m) were used. Three control plots were established in the same environment. An average of three *Schima superba* and five *Pinus massoniana* were growing in each plot. The height and diameter at breast height for each species in the control and drought plots are listed in Table S1. The plots were established in theW middle of the forests, and we attempted to select trees in the middle of the plots.

### CO_2_ response curve

We measured the foliar gas exchange of two species (*Schima superba* and *Pinus massoniana*) using a portable photosynthesis system (LI-6400, LI-COR Inc., USA) during the growing season (early September) of 2012 (Figure S2). Measurements were performed with two portable photosynthesis systems on clear sunny days between 8:00 a.m. to 2:00 p.m. Twelve individuals (4 individuals per plot) for each species were measured in each treatment (control and drought plots). All measurements were performed on fully expanded leaves with no signs of senescence or immaturity. Branches with sun-facing leaves were excised from the middle of the crown with the help of a lopper (3 m) affixed to a bamboo shoot (approximately 10 m) (Figure S3) and then immediately stored in a bottle with fresh water. The gas exchange measurements were conducted soon after branch excision. The time period from branch excision to the completion of the measurement was typically less than 30 min. Typical *A*_n_/*C*_i_ curves (*A*_n_ versus the calculated intercellular CO_2_ concentrations, *C*_i_) were measured at the ambient CO_2_ concentration (*C*_a_) (ranging from 50 to 1400 μmol mol^−1^). The *C*_a_ was lowered stepwise from 400 to 50 μmol mol^−1^ and then increased again from 50 to 1400 μmol mol^−1^, with a total of 10 points. Photosynthesis was induced for 10 min at the saturating photosynthetically active photon flux density (PPFD) (1500 μmol photons m^−2^ s^−1^) at a given leaf temperature (25 °C). The CO_2_ concentrations in the cuvette were controlled using an injector system (LI-6400-01, LI-COR Inc.) that used a CO_2_ mixer and compressed CO_2_ cartridges. The PPFD was provided by the red/blue LED light source built into the foliar cuvette (LI-6400-02B, LI-COR Inc.) and was calibrated against an internal photodiode. The average value of the air temperature on the measurement days was 24.1 °C. The leaf temperature in the cuvette, which was controlled by the thermoelectric cooling elements of the Li-6400, was 25 °C. The cuvette was sealed with plasticine to prevent leakage. We placed twelve needles of *Pinus massoniana* side by side into a 2×3 cm sealed cuvette. The cuvette was sealed with plasticine to prevent leakage^65^,^66^.

*A*_n_/*C*_i_ curves were fitted to estimate the *V*_cmax_, *J*_max_, *TPU* and *g*_m_ using spreadsheet-based software by minimizing the root mean square error (RMSE) of each curve^67^. The *g*_s_ (mmolH_2_O m^−2^ s^−1^) was initially measured with the Li-6400. Due to the differences in diffusion coefficients between water vapor and CO_2_, the stomatal conductance to H_2_O was 1.6-fold higher than the stomatal conductance to CO_2_^68^. To achieve consistency with the *g*_m_, we converted the *g*_s,w_ to *g*_s,c_. The *A*_n_ measured at the 400 μmol mol^−1^ CO_2_ concentration and 25 °C leaf temperature from each *A*_n_/*C*_i_ curve was used to track the differences between treatments for each species. The total conductance (*g*_tot_) was calculated from the sum of the *g*_s_ and *g*_m_.

### Light response curve

The light response curves of *Schima superba* and *Pinus massoniana* were measured using the LI-6400 after each *A*_n_/*C*_i_ curve measurement. The PPFD was sequentially lowered from 1800 to 0 μmol m^−2^ s^−1^, with a total of 14 points. During each measurement, the CO_2_ concentration was maintained at 400 μmol mol^−1^, and the leaf temperature was maintained at 25 °C.

A non-rectangular hyperbola model^69^ solved for its negative root was used to describe the light response curves. In our study, the leaf maximum apparent quantum yield of CO_2_ uptake (AQY, μmol CO_2_ m^−2^s^−1^) and *R*_d_ (μmol CO_2_ m^−2^ s^−1^) were derived from the light response curve. The gross CO_2_ assimilation (*A*_g_) was calculated by adding *R*_d_ to *A*_n_.

### Soil water content and leaf chemical characteristics

The gravimetric soil water content (SWC) was measured at the depth of 0–20 cm in the field. Soil samples from each plot were placed into aluminum boxes and then dried in an oven at 106 °C for 24 h. The SWC in this study was expressed as follows:


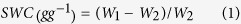


where W_1_ is the sample fresh weight, and W_2_ is the sample dry weight. Finally, eight soil moisture contents were averaged to represent the water content of each plot.

The foliage covered in the cuvette during the gas exchange measurements was used to measure the leaf C and N concentrations (*C*_area_ and *N*_area_) with respect to area. Foliage was removed from branches after the gas exchange measurements, and then the area was measured with an area meter (LI-3100, Li-Cor Inc.). Foliage samples were dried at 65 °C for 48 h, and the leaf characteristics were measured with a CN analyzer. The leaf C/N ratio with respect to area was calculated using the leaf C and N concentrations. The specific leaf area (SLA) was calculated based on the measurements of the leaf area and dry mass.

### Data analyses

The relative limitation to assimilation imposed by the stomatal conductance (*S*_L_), mesophyll conductance (*MC*_L_) and biochemical processes (*B*_L_) were separated using the approach^12^ proposed by Grassi and Magnani (2005). This approach makes it possible to compare relative limitations to assimilation, which partitions photosynthetic limitations into components related to stomatal conductance, mesophyll conductance, and leaf biochemical characteristics. The non-stomatal limitation (*NS*_L_) was defined as the sum of the contribution due to the mesophyll conductance and leaf biochemistry (*NS*_L_ = *MC*_L_ + *B*_L_). The total diffuse limitation (*D*_L_) was the sum of the stomatal and mesophyll conductance components (*D*_L_ = *S*_L_ + *MC*_L_).

The leaf-level intrinsic WUE (iWUE, μmol CO_2_ mmol H_2_O^−1^) was expressed as the ratio of the net CO_2_ assimilation rate versus the stomatal conductance. The NUE (μmol CO_2_ mol N^−1^) for leaf photosynthesis was defined as the ratio of the net photosynthesis rate to the leaf nitrogen content. The AQY derived from the light response curve was the proxy for the leaf-level LUE (μmol CO_2_ μmol Photons^−1^)^70^ in this study.

The drought resistance in this study was expressed as the ratio of the variables in drought plots to those in control plots^71^,^72^ (i.e., variable_drought/variable_control). Values closer to 1 imply greater drought resistance.

We performed independent sample T-tests with a 95% confidence level to examine the differences in each variable among treatments. Previously, the homogeneity of variables was evaluated with Levene’s test (*P* < 0.05). If the homogeneity test failed, the variables were log-transformed or sin-transformed prior to analysis. Regression models were used to determine the relationship between different resource use efficiencies. The general linear model (GLM) was used to test the significance of the slopes of the linear regression among resource use efficiencies. All statistical analyses were performed using SPSS Version 14.0 (SPSS Inc. Chicago, IL, USA).

## Additional Information

**How to cite this article**: Zhou, L. *et al.* Responses of photosynthetic parameters to drought in subtropical forest ecosystem of China. *Sci. Rep.* **5**, 18254; doi: 10.1038/srep18254 (2015).

## Supplementary Material

Supplementary Information

## Figures and Tables

**Table 1:** Soil water content and leaf traits of *Schima superba* and *Pinus massoniana* grown in control and drought plots.

Treatments	SWC (g g^−1^)	*Schima superba*				*Pinus massoniana*			
		SLA (m^2^ kg^−1^)	*C*_area_ (g C m^−2^)	*N*_area_ (g N m^−2^)	Leaf C/N ratio (g g^−1^)	SLA (m^2^kg^−1^)	*C*_area_ (g C m^−2^)	*N*_area_ (g N m^−2^)	Leaf C/N ratio (g g^−1^)
Control	0.21 ± 0.01	10.81 ± 0.58	47.01 ± 2.19	1.67 ± 0.09	28.27 ± 0.62	6.88 ± 0.24	75.84 ± 3.10	2.25 ± 0.09	33.86 ± 1.03
Drought	0.13 ± 0.01	10.85 ± 0.32	46.27 ± 1.35	1.54 ± 0.04	30.08 ± 0.57	7.52 ± 0.41	70.50 ± 4.68	2.10 ± 0.13	33.90 ± 1.37
*p* value	**0.000**	0.959	0.777	0.191	**0.044**	0.262	0.351	0.351	0.982

Note: The drought effects on the soil water content (SWC), specific leaf area (SLA), C concentration (*C*_area_), N concentration (*N*_area_) and Leaf C/N ratio were analyzed for *Schima superba* and *Pinus massoniana* using an independent sample T-test. Significant values (*P* < 0.05) are shown bold (Mean ± SE, N=12).
