# Supplementary material for: Responses of photosynthetic parameters to drought in subtropical forest ecosystem of China
Source: Sci Rep. 2015 Dec 15;5:18254. doi: 10.1038/srep18254 (PMC4678887; doi:10.1038/srep18254)
Supplement: Supplementary Information [file srep18254-s1.pdf]

Title: Responses of photosynthetic parameters to drought in subtropical forest ecosystem of China

Authors: Lei Zhou[1], Shaoqiang Wang[1*], Yonggang Chi[2], Qingkang Li[1], Kun Huang[1], Quanzhou Yu[1]

Affiliations:

[1] Key Laboratory of Ecosystem Network Observation and Modelling, Institute of Geographic Sciences and Natural Resources Research, Chinese Academy of Sciences, Beijing 100101, China

[2] State Key Laboratory of Vegetation and Environmental Change, Institute of Botany, Chinese Academy of Sciences, Beijing 100093, China

[*]Address correspondence to Prof. Shaoqiang Wang, Email: sqwang@igsnrr.ac.cn; Phone: 0086-10-64889666; 0086-10-64889666

Running title: Responses of photosynthetic parameters to drought

Mailing address: 11A, Datun road, Chaoyang District, Beijing, China

Number of tables: 1

Number of figures: 6

Number of reference: 72

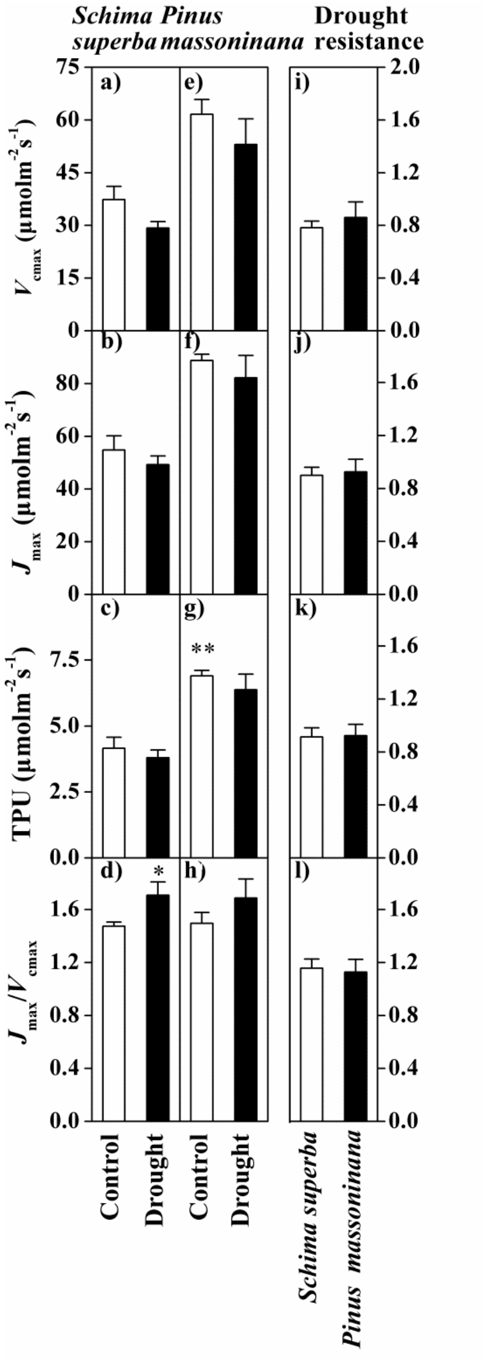

Figure S1 The effect of drought on biochemical processes for the two species. (a, e) The V_{cmax} (the maximum rate of Rubisco carboxylation, μmol m^{-2} s^{-1}), (b, f) J_{max} (the maximum rate of photosynthetic electron transport, μmol m^{-2} s^{-1}), (c, g) TPU (triose-phosphate utilization, μmol m^{-2} s^{-1}) and (d, h) the ratio of J_{max} and V_{cmax} in the control and drought plots of *Schima superba* (a-d) and *Pinus massoniana* (e-h) are shown. The drought resistance of (i) V_{cmax}, (j) J_{max}, (k) TPU and (l) J_{max}/V_{cmax} in *Schima superba* and *Pinus massoniana* is indicated. ANOVA: *, $P<0.05$; **, $P<0.01$; and ***, $P<0.001$.

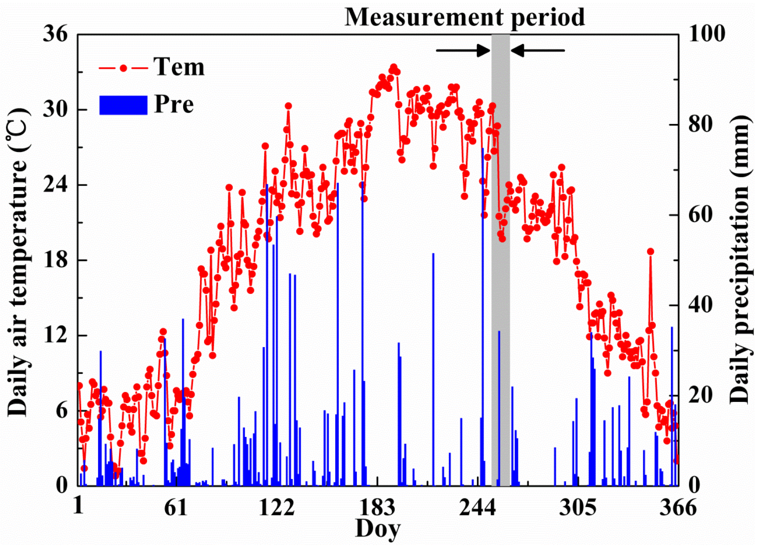

Figure S2 The daily temperature (red points and line) and precipitation (blue bars) in 2012 at the QYZ station and during the measurement period used in our study (gray bar)

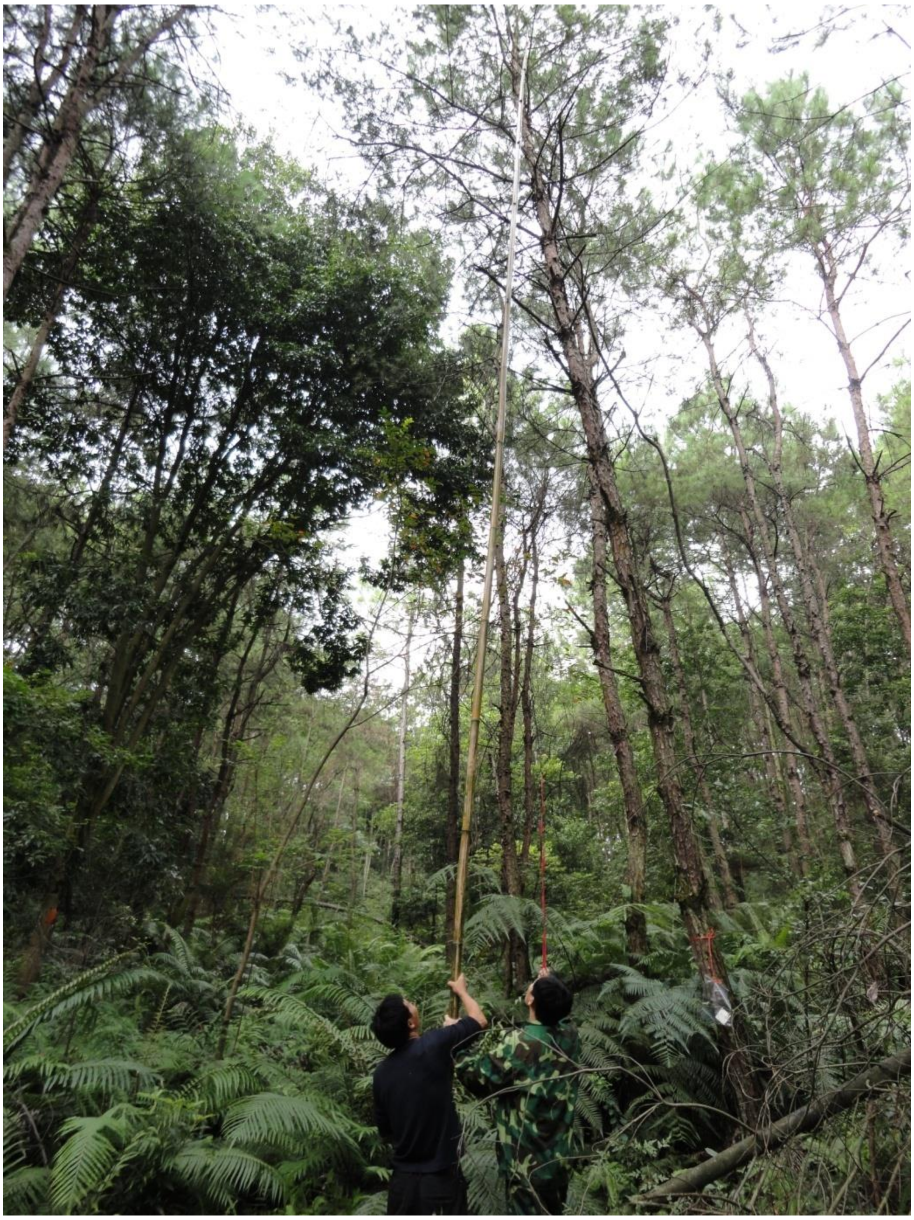

Figure S3 The method used to reach the sample foliage at the tree canopy level. Photo credit: Lei Zhou.

Table S1 The features of trees in the control and drought plots.

Treatments	LAI	Height (m)		Diameter at breast height (cm)	
		Schima superba	*Pinus massoniana*	*Schima superba*	*Pinus massoniana*
Control plots	4.1 ±0.2[a]	15.9 ±0.5[a]	13.6 ±0.4[a]	24.4 ±1.1[a]	20.2 ±1.1[a]
Drought plots	3.5 ±0.3[a]	14.7 ±0.6[a]	14.9 ±0.8[a]	21.9 ±1.7[a]	18.5 ±0.9[a]

Note: Values sharing the same letters are not significantly different at $P < 0.05$
